# Early Health Economic Modeling of Novel Therapeutics in Age-Related Hearing Loss

**DOI:** 10.3389/fnins.2022.769983

**Published:** 2022-03-04

**Authors:** Evie C. Landry, Mirre Scholte, Matthew P. Su, Yvette Horstink, Rishi Mandavia, Maroeska M. Rovers, Anne G. M. Schilder

**Affiliations:** ^1^Division of Otolaryngology-Head and Neck Surgery, St. Paul’s Hospital, BC Rotary Hearing and Balance Centre, University of British Columbia, Vancouver, BC, Canada; ^2^National Institute for Health Research University College London Hospitals Biomedical Research Centre Hearing Theme, London, United Kingdom; ^3^evidENT, Ear Institute, University College London, London, United Kingdom; ^4^Department of Operating Rooms, Radboud University Medical Center, Nijmegen, Netherlands

**Keywords:** Early HTA, novel hearing therapeutics, regenerative hearing therapeutics, age-related hearing loss, hearing loss

## Abstract

**Background:**

Health systems face challenges to accelerate access to innovations that add value and avoid those unlikely to do so. This is very timely to the field of age-related sensorineural hearing loss (ARHL), where a significant unmet market need has been identified and sizeable investments made to promote the development of novel hearing therapeutics (NT). This study aims to apply health economic modeling to inform the development of cost-effective NT.

**Methods:**

We developed a decision-analytic model to assess the potential costs and effects of using regenerative NT in patients ≥50 with ARHL. This was compared to the current standard of care including hearing aids and cochlear implants. Input data was collected from systematic literature searches and expert opinion. A UK NHS healthcare perspective was adopted. Three different but related analyses were performed using probabilistic modeling: (1) headroom analysis, (2) scenario analyses, and (3) threshold analyses.

**Results:**

The headroom analysis shows an incremental net monetary benefit (iNMB) of £20,017[£11,299–£28,737] compared to the standard of care due to quality-adjusted life-years (QALY) gains and cost savings. Higher therapeutic efficacy and access for patients with all degrees of hearing loss yields higher iNMBs. Threshold analyses shows that the ceiling price of the therapeutic increases with more severe degrees of hearing loss.

**Conclusion:**

NT for ARHL are potentially cost-effective under current willingness-to-pay (WTP) thresholds with considerable room for improvement in the current standard of care pathway. Our model can be used to help decision makers decide which therapeutics represent value for money and are worth commissioning, thereby paving the way for urgently needed NT.

## Introduction

Hearing loss currently affects almost 500 million people worldwide and this number is anticipated to rise to 900 million by 2050 as the world’s population ages and the number of individuals with age-related hearing loss (ARHL) increases (Blevins, 2018; WHO, 2018). This type of hearing loss is characterized by a decline in auditory function predominantly due to progressive loss of inner ear sensory hair cells and their synapses to auditory neurons (Yamasoba et al., 2013). Irrespective of its cause and severity, hearing loss can affect people socially, mentally, and physically. Importantly, hearing loss acquired in mid-life has been identified as a major risk factor for dementia (Lin and Albert, 2014). The global economic burden of hearing loss has been estimated at over 750 billion US dollars annually in direct medical costs and productivity losses (WHO, 2018).

The mainstay of treatment for ARHL includes hearing aids for those with mild to moderate hearing loss and cochlear implants for those with severe to profound deafness (WHO, 2018). Although these technologies have improved significantly in recent years, they often fail to meet the needs of those who need them most as they perform poorly in noisy environments and are limited in their ability to improve sound clarity. Therefore, many people choose not to use them (McCormack and Fortnum, 2013). Above all, they do not treat the underlying causes of ARHL or halt progression (McCormack and Fortnum, 2013).

Biotechnology and pharmaceutical companies have identified this unmet market need and have dedicated sizeable effort and investments in the development of novel approaches to treat ARHL (Li, 2017). A better understanding of the genetic and molecular mechanisms underlying hair cell and synaptic loss and their regeneration in preclinical models has led to the discovery of potential therapeutic targets, and the development of a variety of small molecule pharmaceuticals and advanced therapies (Schilder et al., 2018). Some of these novel therapeutics are already at the stage of clinical testing in humans (Schilder et al., 2018).

Because these therapeutics have the potential to drastically change hearing care pathways in the next 5 years, it is crucial to start planning for their implementation (Schilder et al., 2019). Early health economic modeling is an important tool in this process. By providing a better understanding of the likely cost-effectiveness of the novel hearing therapeutics, healthcare systems can use these models to prepare for their adoption while they are still in development and thus optimize patient access and minimize inefficiencies. At the same time, these models can support industry by informing product development, market access, pricing, and can also act as frameworks that can be tailored with data from clinical trials as it becomes available (IJzerman and Steuten, 2011).

This study applies these principles and uses early health economic modeling to assess the potential added value of novel regenerative therapeutics in ARHL compared to the current standard of care.

## Materials and Methods

### Ethical Considerations

Ethics approval was granted by the University College London Research Ethics Committee 12241/001. Informed consent was obtained from all participants.

### Model Overview and Assumptions

**FIGURE 1:**
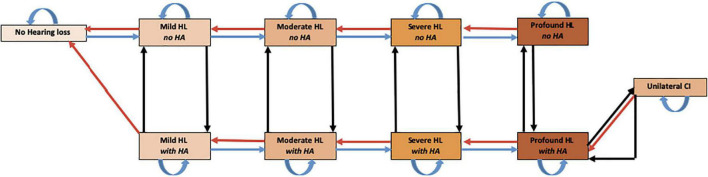
Markov Model of health states used to assess regenerative hearing loss therapeutics. The model starts with a cohort of 50-year-old patients with various degrees of HL. Every cycle subjects could progress to 1 of 11 mutually exclusive disease states including death. Though movement is possible between every state, natural death and all arrows not depicted for simplicity. HL, hearing loss; HA, hearing aid; CI, cochlear implant.

A state-transition model, following the ISPOR-SMDM Best Practice Guidelines, was created using Microsoft Excel (Redmond, Wash) to assess the potential costs and effects of using novel regenerative hearing therapeutics in adults, 50 or older, with ARHL (Roberts et al., 2012). This was compared to the current standard of care, including hearing aids and cochlear implants. Figure 1 shows a representation of the patient’s pathway modeled in the study. Supplemental Digital Content (SDC) 1 contains an in-depth description of hearing loss classification using pure-tone averages (PTA) (Mathers et al., 2000).

The model adopts a cycle length of 1 year and spans the patient’s lifetime until death given the life-long costs and effects of hearing loss. The model is constructed from a healthcare perspective of the National Health Service (NHS) in the United Kingdom and evaluates direct medical costs. A willingness to pay threshold of £20,000 per quality-adjusted life year (QALY) is used to assess the cost-effectiveness (CE) of the intervention (NICE, 2017). Both costs and outcomes are discounted at a 3.5% per annum rate to comply with NICE guidelines (National Institute for Health and Clinical Excellence, 2008). NICE stands for the National Institute for Health and Care Excellence. It is an independent organization which evaluates and provides recommendations of which drugs and treatments are available on the NHS in England. Other key model assumptions can be found in SDC 2. See SDC 3 for a complete list of abbreviations used in the text along with verbal descriptors.

### Model Validation

The model was validated using the AdViSHE validation assessment tool (Vemer et al., 2016). The conceptual model, input data, and model outcomes were tested on face and operational validity by consulting professional stakeholders (*n* = 24) from the pharmaceutical and biotechnology industry, national hearing charities, otolaryngology, audiology, discovery science, and research funding bodies. The model outcomes were cross validated with relevant literature. No other health economic models on ARHL were found for cross-validation. Additionally, the model was verified for inconsistencies by two independent modeling experts.

### Novel Hearing Therapy Pathway

With no regenerative therapeutic for ARHL having been approved for clinical use, we used a hypothetical regenerative therapy to model different efficacy scenarios. Our model focused primarily on regenerative therapeutics and was not targeted toward a specific gene, cell, molecular therapy or method of delivery. In order to estimate the maximum potential benefit of the therapeutic (headroom), the base case scenario assumed a 100% adherence, uptake and efficacy of the therapy with zero costs. This meant that patients with any form of hearing loss recovered to normal hearing after therapy administration. It also assumed that rates of hearing loss progression in subsequent years were unaffected, meaning that patients could once again develop age-related hearing loss. The model assumed that all patients were eligible for both existing and novel strategies and could receive either a hearing aid, a cochlear implant, or the novel therapy as they developed hearing loss in the model.

### Model Parameters

#### Transition Probabilities

Transition probabilities were derived from published literature and expert opinion (Tables 1A,B). The model population included five different age groups: 50−59, 60−69, 70−79, 80−89, and 90 and over, with different transition probabilities for progression of age-related sensorineural hearing loss (Table 1A). Age-varying all-cause mortality rates were also incorporated in the model using data from the UK Office for National Statistics (2018b). Although several papers suggested an association between HL in older adults and increased mortality rates, it was felt the evidence was incomplete and it was therefore not incorporated into our model (Fisher et al., 2014; Contrera et al., 2015; Schubert et al., 2016).

**TABLE 1:** Transition probabilities.

1A | Summary of annual transition probabilities of hearing status (Chao and Chen, 2008).*						
Initial hearing status	Age	Status in next cycle				

	Years	Normal	Mild HL	Moderate HL	Severe HL	Profound HL
Normal	50–59	9.57E−01	4.11E−02	1.37E−03	1.99E−07	1.99E−07
	60–69	9.67E−01	3.24E−02	3.30E−04	6.28E−06	6.28E−06
	70 and over	9.64E−01	3.55E−02	5.60E−04	4.15E−06	4.15E−06
Mild HL	50–59	0	9.31E−02	6.93E−02	2.00E−05	2.00E−05
	60–69	0	9.79E−02	1.98E−02	5.70E−04	5.70E−04
	70 and over	0	9.70E−02	2.91E−02	3.30E−04	3.30E−04
Moderate HL	50–59	0	0	9.99E−01	4.30E−04	4.30E−04
	60–69	0	0	8.90E−01	5.48E−02	5.48E−02
	70 and over	0	0	9.56E−01	2.20E−02	2.20E−02
Severe HL	50–59	0	0	0	1.00E + 00	4.30E−04
	60–69	0	0	0	9.45E−01	5.48E−02
	70 and over	0	0	0	9.78E−01	2.20E−02

1B | **Transition probabilities**.						

**Parameters**		**Mean**		**Distribution**	**References**	

**Baseline parameters**						
Discount rate		3.50%		0–6%	NICE guidelines (NICE, 2017)	
Transition probabilities						
Probabilities of hearing status		See Table 1A		Dirichlet	Chao and Chen, 2008	
Probabilities of death		See lifetables		–	UK Office for National Statistics, 2018b	
**Probability of functional imp.**						
Normal HL		0.18		Beta	Choi et al., 2016	
Mild HL		0.22		Beta	Choi et al., 2016	
Moderate HL		0.26		Beta	Choi et al., 2016	
Severe HL		0.26		Beta	Choi et al., 2016	
Profound HL		0.26		Beta	Choi et al., 2016	
**Probabilities of receiving HA**						
Mild HL		0.30		Dirichlet	EuroTrak, 2018	
Moderate HL		0.52		Dirichlet	EuroTrak, 2018	
Severe HL		0.71		Dirichlet	EuroTrak, 2018	
Profound HL		0.71		Dirichlet	EuroTrak, 2018	
Profound HL with 1 CI		0.58		Dirichlet	Fielden and Kitterick, 2016	
**Probability of needing a CI**						
Profound HL		0.60		Dirichlet	Expert opinion, Bond et al., 2009	
**Probability of not using HA**						
Mild HL		0.13		Dirichlet	Chao and Chen, 2008	
Moderate HL		0.30		Dirichlet	Chao and Chen, 2008	
Severe HL		0.30		Dirichlet	Chao and Chen, 2008	
Profound HL		0.30		Dirichlet	Expert opinion, Bond et al., 2009	
Profound HL with CI		0.03		Dirichlet	Fielden and Kitterick, 2016	
**Probability of non-use/device failure in CI**						
Prob of CI non-use/failure		0.03		Dirichlet	NICE, 2007	
Major complication		Year 1: 0.04		Beta	Bond et al., 2009	
					Farinetti et al., 2014	
					Stamatiou et al., 2011	

*HL, Hearing Loss; HA, Hearing aid; CI, Cochlear Implant.*

*Transition probabilities are based on the average for both men and women. Dirichlet distributions were applied for all parameters.

#### Outcomes

Health effects were measured using QALYs that factored both length and quality of life into a single measure (SDC 6). Preference based utility measures of health-related quality of life were obtained from published literature (Table 2). Due to the poor sensitivity of the European quality of life five dimension (EQ-5D) in sensory disorders such as hearing loss, the Health utilities index mark III (HUI-3) was used (Longworth et al., 2014; Yang Y. et al., 2015). The utility score provides a summary index of health-related quality of life on a zero to one scale. Functional impairment was incorporated in all hearing loss states, except for the cochlear implant state due to lack of available data. Functional impairment was defined as difficulties in activities of daily living (ADLs) correlated to pure-tone averages (Choi et al., 2016). We assumed the utility of being functionally impaired was equal to the lowest value of the lower bound of the parameters’ 95% confidence interval (CI). A measure of functional impairment was included at the request of our expert stakeholders. Stakeholders felt incorporating functional impairment to a percentage of hearing health states better reflects reality.

**TABLE 2:** Utilities.

Utilities	Value	Range	Tool	Distribution	References
**HL utilities**					
Utility of normal hearing	0.95	SE 0.08	HUI-3	Beta	Linssen et al., 2015
Utility of FI in normal hearing	0.79		HUI-3	Beta	Linssen et al., 2015
Utility of mild HL	0.80	SE 0.03	HUI-3	Beta	Linssen et al., 2015
Utility of FI in mild HL	0.74		HUI-3	Beta	Linssen et al., 2015
Utility of moderate HL	0.73	SE 0.03	HUI-3	Beta	Linssen et al., 2015
Utility of FI in moderate HL	0.67		HUI-3	Beta	Linssen et al., 2015
Utility of severe HL	0.73	SE 0.03	HUI-3	Beta	Linssen et al., 2015
Utility of FI in severe HL	0.67		HUI-3	Beta	Linssen et al., 2015
Utility of profound HL	0.46	SE 0.21	HUI-3	Beta	Arnoldner et al., 2014
Utility of FI in profound HL	0.26		HUI-3	Beta	Arnoldner et al., 2014
**HL utilities with HA**					
Utility of mild HL with HA	0.89		HUI-3	Beta	Linssen et al., 2015
Utility of FI in mild HL with HA	0.83		HUI-3	Beta	Linssen et al., 2015
Utility of moderate HL with HA	0.90		HUI-3	Beta	Linssen et al., 2015
Utility of FI in moderate HL with HA	0.84		HUI-3	Beta	Linssen et al., 2015
Utility of severe HL with HA	0.90		HUI-3	Beta	Linssen et al., 2015
Utility of FI in severe HL with HA	0.84		HUI-3	Beta	Linssen et al., 2015
Utility of profound HL with HA	0.64		HUI-3	Beta	Linssen et al., 2015
Utility of FI in profound HL with HA	0.43		HUI-3	Beta	Linssen et al., 2015
**HL utility with CI**					
Utility of using a CI	0.61	SE 0.19	HUI-3	Beta	Arnoldner et al., 2014

*HL, Hearing Loss; HA, Hearing aid; CI, Cochlear Implant; FI, Functional Impairment; SE, Standard Error.*

#### Costs

Direct medical costs were assessed in 2018 Pounds (£) and adjusted for inflation using the consumer price inflation indices produced by the World Bank and the UK Office for National Statistics (SDC 7) (UK Office for National Statistics, 2018a; World Bank Group, 2018). Unit costs were derived from the literature, NHS reference cost databases, and institutional level costs from an NHS trust. SDC 8 contains a more in-depth breakdown of each unit cost. These cost estimates reflect what the NHS would cover and exclude any non-medical, opportunity, and patient incurred costs. Given the assumption that patients in the normal hearing health state are asymptomatic allows us to also assume that they will not incur any associated health care costs. Cost of the regenerative hearing therapy was set to be £0 for the headroom analysis. See Table 3 for all costs used in the model.

**TABLE 3:** Costs.

Description	Unit costs (2018£)	Distribution	References
**Direct medical costs**			
Cost of novel hearing loss therapeutic			
Novel therapeutic cost	£0	–	–
**Cost of hearing aids**			
Monaural pathway	£275	Gamma*	NICE, 2017
Binaural pathway	£380	Gamma*	NICE, 2017
Proportion of binaural HA users	0.58	Beta	NICE, 2017
Cost HA aftercare	£26	Gamma*	NICE, 2017
Cost of hearing evaluation for HA	£54	Gamma*	NICE, 2017
Proportion of patients receiving HL assessment without getting HAs Mild HL Moderate HL or worse	0.30 0.05	Beta Beta	Davis et al., 2007 Davis et al., 2007
**Cost of CI**			
Unilateral cochlear implant cost	£22, 919	Gamma*	NHS Improvement, 2018
Presurgical CI candidacy costs	£5,308	Gamma*	NHS trust costs (UK Cochlear Implant Study Group, 2004)
Proportion of patients receiving CI assessment without getting a CI	0.40	Beta	Expert opinion
**Post implantation costs**			
Maintenance costs in year 1	£6,617	Gamma*	NHS trust costs (UK Cochlear Implant Study Group, 2004)
Maintenance costs in year 2+	£945	Gamma*	NHS trust costs (UK Cochlear Implant Study Group, 2004)
Processor upgrade every 5 years	£5,445	Gamma*	NHS trust costs (Bond et al., 2009)
Cost of major complication	£10,292	Gamma*	UK Cochlear Implant Study Group, 2004

HL, Hearing Loss; HA, Hearing aid; CI, Cochlear Implant; * Varied by 10% to account for pricing differences across the United Kingdom.

#### Analysis

A hypothetical cohort of 1,000 patients entered the model to determine the mean expected costs and effects (QALYs) per patient. Three different but related analyses were performed: (1) headroom analysis, (2) scenario analyses, and (3) threshold analyses. All results were obtained using probabilistic modeling.

The headroom analysis explored the maximum potential value of a novel regenerative therapeutic for ARHL. The headroom approach to therapeutic developments relies on the estimation of value-based price-ceilings. Such price-ceilings estimate the commercial opportunities for new products in health care systems (Girling et al., 2015). To delineate the ceiling-price, the initial scenario assumed that the therapeutic was 100% effective, meaning that all patients with any level of hearing loss returned to normal hearing (PTA: ≤25dB) and was delivered at no cost. Six scenario analyses were then developed to explore how more realistic scenarios influenced costs and effects: (1) limiting the therapeutic to different HL severities (all HL severities, only moderate HL or worse, only severe HL or worse, etc.) or (2) varying the degree of recovery (all recover to normal hearing, recovery by 1 health state such as recovering from severe to moderate HL or by 2 health states such as recovering from severe to mild HL, etc.). Threshold analysis was then used to determine the ceiling price of the novel therapeutics at different disease severities and levels of effectiveness.

Probabilistic sensitivity analyses (PSA) with 10,000 iterations were used to obtain results for all scenarios to account for uncertainty around parameter estimates (Baio and Dawid, 2015). See Tables 1–3 for distributions applied to each parameter. Given that ranges for costs were not explicitly stated in the literature, the gamma distributions were varied by 10% to account for pricing variations across the United Kingdom. 95% confidence intervals (95% CIs) of probabilistic results were calculated by the percentile method in Excel (Elias, 2015).

Results are presented in incremental net monetary benefits (iNMB). The iNMB represents the added value of an intervention compared to the current standard of care, in monetary terms. iNMB is calculated by using the following formula: iNMB = (QALY_n_ × threshold value – Costs_n_) – (QALY_*c*_ × threshold value – Costs_c_), where *n* = novel therapeutic, NICE threshold value = £20,000/QALY and, *c* = current treatment (NICE, 2017). Higher incremental NMBs equate to greater potential room for improvement. Positive iNMBs indicate that novel therapeutics are potentially cost-effective as compared to the standard of care pathway.

## Results

### Headroom Analysis

Table 4 summarizes the results of the headroom analysis and shows that total costs and QALYs per patient in the standard care pathway are £4,462 [£3,262–£5,663] and 15.59 [15.09–16.09], respectively. The total costs and QALYs per patient for a perfect novel hearing therapeutic strategy are £11 [£5–£17] and 16.37 [15.67–17.06], respectively. This yields potential savings of £4,451 [£3,254–£5,648] and QALY gains of 0.78 [0.37–1.19] per patient. The iNMB of a perfect, zero cost, novel regenerative hearing therapeutic in ARHL is £20,017 [£11,299–£28,737].

**TABLE 4:** Headroom and scenario analysis results.

Scenario	Recovery	Severity	Cost ST current	Cost NT new	QALY ST current	QALY NT new	iNMB
Headroom	To normal	All	£4,262 [3,262, 5,663]	£11 [5, 17]	15.59 [15.09, 16.09]	16.37 [15.67, 17.06]	£20,018 [11,299, 28,737]

			**Incremental costs**		**Incremental QALYs**		

1	To normal	All	−£4,451 [−5,648, −3,254]		0.78 [0.37, 1.19]		£20,018 [11,299, 28,737]
2	Back by 1 state	All	−£2,151 [−2,787, −1,515]		0.27 [0.16, 0.38]		£7,521 [4,884, 10,158]
3	Back by 2 states	All	−£2,582 [−3,355, −1,809]		0.33 [0.19, 0.48]		£9,265 [5,861, 12,670]
4	To normal	M/S/P	−£3,920 [−5,009, −2,824]		0.46 [0.29, 0.63]		£13,127 [8,928, 17,326]
5	To normal	S/P	−£3,185 [−4,165, −2,205]		0.29 [0.18, 0.41]		£9,046 [5,957, 12,135]
6	To normal	P	−£2,923 [−3,789, −2,056]		0.25 [0.15, 0.35]		£7,896 [5,249, 10,544]

*ST, standard therapy pathway; NT, novel therapeutic pathway; M, moderate; S, severe; P, profound.*

*All scenarios assumed the novel therapeutic to be 100% effective and cost £0 95% confidence intervals are shown in brackets.*

### Scenario Analyses

The results demonstrated that compared to the headroom scenario (scenario 1), changing the recovery rates by only one (scenario 2) or two (scenario 3) hearing health states lowers the iNMB to £7,521 [£4,884–£10,158] and £9,265 [£5,861–£12,670], respectively (Table 4). Since the assumed drug cost is zero due to the headroom scenario, limiting treatment to patients with more severe degrees of hearing loss (scenarios 4–6) decreased the iNMB, owing to the fact that only a subgroup of the model’s population had more severe forms of hearing loss. This resulted in only a proportion of the total population being treated in this scenario which decreased the overall QALY gains and resulted in less cost savings.

### Threshold Analyses

**FIGURE 2:**
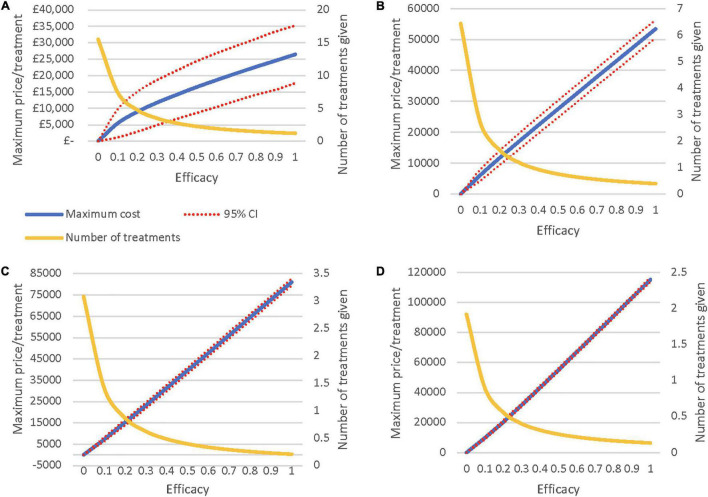
**(A)** Maximum Price when treating all severities of HL. **(B)** Maximum Price when treating moderate HL or worse. **(C)** Maximum Price when treating severe HL or worse. **(D)** Maximum Price when treating only profound HL.

The threshold analyses illustrate the ceiling prices of the novel regenerative hearing therapeutics with differing rates of effectiveness and hearing loss severities (Figures 2A–D). The lines in the graphs represent an iNMB of £0, identifying (1) the potential maximum price for each level of effectiveness, (2) the 95% confidence intervals, and (3) the average number of treatments given per person depending on the efficacy of the therapy. For example, if treatment is restricted to patients with moderate hearing loss or worse (Figure 2B), the ceiling price at an efficacy of 50% will be around £27,500/per person. See SDC 9 for a graphical comparison of threshold analyses results.

## Discussion

### Summary of Key Findings

The headroom scenario yields an iNMB of £20,017 [£11,299–£28,737] compared to the current standard of care and show that novel regenerative hearing therapeutics have potential room for improvement in terms of both improved patient outcomes with QALY gains of 0.78 [0.37–1.19] and cost savings of £4,451 [£3,254–£5,648] per patient. Scenario analyses demonstrate that the iNMB increases with more effective therapeutics and more widespread eligibility criteria. Threshold analysis suggests that the ceiling price of the therapeutic is greater with severe forms of hearing loss compared to less severe states. Both scenario and threshold analyses demonstrate wide confidence intervals which reflects the uncertainty surrounding results.

### Strengths

This study shows that there is significant potential room for improvement in the current standard care pathway for patients with ARHL and that novel regenerative hearing therapeutics could become cost-effective in the NHS. This model can be used by both industry and policy makers to evaluate: (1) the maximum price of a novel regenerative therapeutic in ARHL for different levels of effectiveness, (2) the minimum effectiveness required at each pricing target for the therapeutic to remain cost-effective, and (3) the impact of limiting treatment to certain subgroups of patients with hearing loss. Exploring the ceiling price of novel therapeutics at maximum efficacy will also allow pharmaceutical companies to perform feasibility estimates on the potential to recoup research and development (R&D) costs (IJzerman and Steuten, 2011; Markiewicz et al., 2016).

Given that one in five people in the United Kingdom are estimated to be affected by a hearing disorder by 2035, means that the implementation of novel hearing therapeutics will bring about substantial change to health systems (No author list, 2016). The development of this model, before a therapeutic has entered the market, will facilitate informed decision making and will increase the likelihood of developing cost-effective novel regenerative hearing therapeutics. As these novel hearing therapeutics enter into clinical trial, this research will help commissioners and policy makers to make difficult decisions on which therapeutics provide value for money are worth funding, thereby paving the way for revolutionary and urgently needed hearing therapeutics.

### Limitations

Despite the robustness of our results to a variety of probabilistic sensitivity analyses, this study is subject to limitations; the first of which arises from our limited scientific understanding of ARHL. In addition to ARHL not being well understood, its association and interrelationship with age related neurocognitive decline further complicates our understanding. ARHL is not a single disease entity but a symptom of a range of underlying disease mechanisms and etiologies, resulting in an equal range of potential therapeutic targets within the inner ear and auditory pathways (Yamasoba et al., 2013; Nakagawa, 2014). As such there will be no single cure for hearing loss; in order to develop targeted therapeutics for hearing disorders in specific patient populations more detailed pheno-and genotyping of hearing loss patients will be required (Yang C. H. et al., 2015; Le Prell et al., 2016). For our model, this means that our results likely represent an overestimation of the target population with ARHL that will be eligible to receive and benefit from regenerative hearing therapeutics in the future. Despite these limitations, our model reflects our current understanding and uses the best available data on progression and utilities for ARHL.

Another limitation is that we did not include drug safety profiles for novel hearing therapeutics due to their hypothetical nature. This model assumes that along with being perfectly effective, the novel hearing therapies are free of adverse events which overestimates their potential added value. Finally, this study focused on direct medical costs incurred by the NHS and did not include indirect medical costs associated with hearing loss in either the standard of care or novel hearing therapeutic arms (NICE, 2017). This has likely led to an underestimation of the costs that NICE requires for decision making. Additionally, the PSA’s accuracy is inherently limited by the nature of modeling a hypothetical novel hearing therapeutic and by arbitrary variations in costs set at 10%.

### Future Research

Consultation with our panel of expert stakeholders revealed strong support to move away from the classic classification of hearing loss using pure-tone averages to a classification scheme that incorporates functional impairment. Hearing in Noise Testing (HINT) was proposed as a potential solution moving forward. In order to construct new models using HINT, as opposed to pure-tone averages, further research will be required to delineate both ARHL transition probabilities and utilities in identified HINT states.

A growing body of evidence suggests that the use of EQ-5Ds, as recommended by NICE, may not be appropriate for all health conditions such as hearing disorders and vision loss due to the poor responsiveness of the measure to detect change (Longworth et al., 2014). Our review of the literature highlights the lack of consensus amongst studies on the best utility estimates to employ but did identify the HUI-3 as the most commonly used measure in health economic evaluations relating to hearing loss and was therefore employed in our model (Longworth et al., 2014; Yang Y. et al., 2015). To enhance comparability across studies, either the use of HUI-3s should be standardized or an EQ-5D ‘bolt-on’ for hearing should be incorporated to improve its sensitivity (Yang Y. et al., 2015).

Finally, this study not only has the capacity to inform current R&D decisions, but also to accelerate decisions in later stages of development once better therapeutic efficacy estimates have been established. As an increasing number of these therapeutics enter into clinical trials, it will be important to incorporate new evidence as it becomes available. This will improve the quality and reliability of the results, allowing for lifecycle HTA of the therapeutics. It will also be vital to conduct similar early health technology assessments in other therapeutic areas of hearing loss such as sudden sensorineural hearing loss and noise and drug induced hearing loss as the field expands (Hartz and John, 2008; Markiewicz et al., 2016; Mandavia et al., 2020).

## Conclusion

This study presents the first early health economic model for regenerative hearing therapeutics in ARHL and demonstrates a large potential room for improvement in the current care pathway. Novel regenerative hearing therapeutics for ARHL could become cost-effective under current willingness-to-pay thresholds. This model can be used by policy makers and industry to support the development of cost-effective therapies with the largest potential to provide added value to society and will help accelerate the introduction of ground-breaking novel hearing therapeutics in the NHS and around the world.

## Data Availability Statement

The original contributions presented in the study are included in the article/Supplementary Material, further inquiries can be directed to the corresponding author.

## Ethics Statement

The studies involving human participants were reviewed and approved by the University College London Research Ethics Committee 12241/001. The patients/participants provided their written informed consent to participate in this study.

## Author Contributions

EL designed, collected data, performed the modeling, and wrote the main manuscript. MS, MPS, and YH designed, collected data, performed the modeling, and provided interpretive analysis. RM, MR, and AS provided interpretive analysis and critical revisions. All authors discussed the results and implications and commented the manuscript at all stages.

## Conflict of Interest

AS advises biotech and pharmaceutical companies in the hearing field on the design and delivery of clinical trials. The remaining authors declare that the research was conducted in the absence of any commercial or financial relationships that could be construed as a potential conflict of interest.

## Publisher’s Note

All claims expressed in this article are solely those of the authors and do not necessarily represent those of their affiliated organizations, or those of the publisher, the editors and the reviewers. Any product that may be evaluated in this article, or claim that may be made by its manufacturer, is not guaranteed or endorsed by the publisher.
