# Supplementary material for: Early Health Economic Modeling of Novel Therapeutics in Age-Related Hearing Loss
Source: Front Neurosci. 2022 Mar 4;16:769983. doi: 10.3389/fnins.2022.769983 (PMC8930912; doi:10.3389/fnins.2022.769983)
Supplement: Supplementary file 1 [file Data_Sheet_1.zip › SDC 1.DOCX]

**SUPPLEMENTAL DIGITAL CONTENT**

**SDC 1: WHO Classification of Hearing Loss**

The World Health Organization (WHO) classification of hearing loss using pure tone averages (PTA) used to define hearing health states in the model.

**Table 1.** WHO Classification of Hearing Loss (1)

| PTA (0.5- 4kHz) | Verbal descriptor |
| --- | --- |
| ≤ 25 dB HL | Normal |
| 26- 40 dB HL | Mild |
| 41-60 dB HL | Moderate |
| 61-80dB HL | Severe |
| ≥ 81 dB HL | Profound |
