# Supplementary material for: Early Health Economic Modeling of Novel Therapeutics in Age-Related Hearing Loss
Source: Front Neurosci. 2022 Mar 4;16:769983. doi: 10.3389/fnins.2022.769983 (PMC8930912; doi:10.3389/fnins.2022.769983)
Supplement: Supplementary file 1 [file Data_Sheet_1.zip › SDC 2.DOCX]

**SDC 2: Key Model Assumptions**

**Table 2.** Model Assumptions

| **Model assumptions** |
| --- |
| - The model population includes both men and women and is comprised of five different age groups: 50-59, 60-69, 70-79 and 80-89 and 90 and over, most of which had distinct transition probabilities for progression of hearing loss. - Progression rates of hearing loss between sexes was assumed to be the same for the purpose of this study. Average age-adjusted probabilities for both males and females were calculated. - For patients with binaural hearing loss it was assumed that they would be placed in the health state equivalent to the better hearing ear i.e. if a patient suffered from mild hearing loss in one ear and moderate hearing loss in the other, they were placed in the mild hearing loss health state (36). - Due to lack of available data, annual hearing loss transition probabilities were assumed to have the same rates from age 70 and over in the model. - The model starts with a cohort of 50-year-old individuals with various degrees of hearing loss based on UK populational estimates (SDC 4 contains further detail) (44). - Individuals were able to progress to any worse hearing health state within one cycle i.e. a patient with mild hearing loss could progress to a severe hearing loss state within a single cycle. These arrows were not represented in the model diagram for simplicity. - Similarly, patients treated with regenerative hearing therapies could recover hearing to any better hearing health state i.e. a patient with severe hearing loss could recover to normal hearing within a single cycle. These arrows were not represented in the model diagram for simplicity. - The model used age-adjusted all-cause mortality rates. Although several papers suggest an association between HL in older adults and increased mortality rates, the authors felt that further research was necessary before robust conclusions on this association could be established 16-18). - Whilst novel hearing therapies are capable of otoprotection, neuromodulation, restoration and regeneration, this initial modelling approach focused primarily on regeneration. - Given that a headroom methodology was employed, the baseline model assumed 100% adherence, uptake and efficacy of the novel therapy. This meant that patients with any form of hearing loss recovered to normal hearing after therapy administration. - The model assumed that all patients with hearing loss were eligible to receive novel hearing therapies. - Although patients had full recovery to normal hearing, it was assumed that the rates of hearing loss progression in subsequent years was unaffected and patients could once again develop hearing loss. - A drug safety profile of the novel therapy was not included in the model. - Hearing aid duration (that is, the time difference between provision of a hearing aid and it needing to be replaced) was conservatively set at 3 years (34). The cost of a new hearing aid was averaged over 3 years to obtain annual costs. - Patients fitted with hearing aids could move back and forth between using or not using their hearing aids (non-compliance). - Hearing aids could either be fitted monaurally or binaurally. - Complications associated with the use of hearing aids were not included. - Only unilateral CIs were considered in this population given the rarity of bilateral CI indications (47). - Although indications are expanding with regards to CI candidacy, only patients with profound hearing loss were eligible to receive CIs in this model. - Cochlear implant external processor duration (that is the time difference between provision of a cochlear implant and it needing to be replaced) was set at 5 years (47). The cost a replacement CI sound processor was averaged over 5 years to obtain annual costs. - Major complications after CI occurring within the first year after surgery were included in the model. A major complication is defined by the necessity of revision surgery under general anesthetic (47). - Parameters found in the literature which were expressed in rates were converted to probabilities;   SDC 5 contains the formula that was applied. |
