# Supplementary material for: Early Health Economic Modeling of Novel Therapeutics in Age-Related Hearing Loss
Source: Front Neurosci. 2022 Mar 4;16:769983. doi: 10.3389/fnins.2022.769983 (PMC8930912; doi:10.3389/fnins.2022.769983)
Supplement: Supplementary file 1 [file Data_Sheet_1.zip › SDC 3.DOCX]

**SUPPLEMENTAL DIGITAL CONTENT**

**SDC 3: Abbreviations**

Below is a summary of all abbreviations and acronyms used in the paper.

**Table 1.** Abbreviations

| Abbreviation | Verbal descriptor |
| --- | --- |
| ARHL | Age-related sensorineural hearing loss |
| NT | Novel hearing therapeutics |
| NHS | National Health Service |
| iNMB | Incremental Net Monetary Benefit |
| QALY | Quality-adjusted life-year |
| WTP | Willingness to Pay |
| SNHL | Sensorineural Hearing loss |
| SDC | Supplemental Digital Content |
| PTA | Pure-tone averages |
| CE | Cost-Effectiveness |
| HL | Hearing Loss |
| ADL | Activities of daily living |
| CI | Confidence Interval |
| PSA | Probabilistic sensitivity analyses |
| R&D | Research and Development |
| NICE | The National Institute for Health and Care Excellence |
| HINT | Hearing in Noise Testing |
| HTA | Health Technology Assessment |
| UK | United Kingdom |
| WHO | World Health Organization |
