# Supplementary material for: Early Health Economic Modeling of Novel Therapeutics in Age-Related Hearing Loss
Source: Front Neurosci. 2022 Mar 4;16:769983. doi: 10.3389/fnins.2022.769983 (PMC8930912; doi:10.3389/fnins.2022.769983)
Supplement: Supplementary file 1 [file Data_Sheet_1.zip › SDC 4.DOCX]

**SDC 4: Populational Distribution based on UK population**

Estimates are drawn from a paper by Davis et al. that uses PTA thresholds to define the UK populational distribution of hearing loss in various age categories; these percentages were then applied to our theoretical cohort of 1000 patients in the model (44).

**Table 1.** Populational distribution (for 1000 individuals) (44)

| Initial Hearing Status  (years) | Normal hearing | Mild HL | Moderate HL | Severe HL | Profound HL |
| --- | --- | --- | --- | --- | --- |
| 50-59 | 882 | 90 | 22 | 5 | 1 |
