# Supplementary material for: Early Health Economic Modeling of Novel Therapeutics in Age-Related Hearing Loss
Source: Front Neurosci. 2022 Mar 4;16:769983. doi: 10.3389/fnins.2022.769983 (PMC8930912; doi:10.3389/fnins.2022.769983)
Supplement: Supplementary file 1 [file Data_Sheet_1.zip › SDC 6.DOCX]

**SDC 6: QALY Formula**

**QALY= LYG * Utility value**

QALY= Quality-adjusted life year

LYG= Life year gained
