# Supplementary material for: Early Health Economic Modeling of Novel Therapeutics in Age-Related Hearing Loss
Source: Front Neurosci. 2022 Mar 4;16:769983. doi: 10.3389/fnins.2022.769983 (PMC8930912; doi:10.3389/fnins.2022.769983)
Supplement: Supplementary file 1 [file Data_Sheet_1.zip › SDC 7.DOCX]

| **SDC 7: Inflation Indices and Currency Conversion** | | | | | | | | | | | | | | | | | | | | | | | | | | | | | |  |
| --- | --- | --- | --- | --- | --- | --- | --- | --- | --- | --- | --- | --- | --- | --- | --- | --- | --- | --- | --- | --- | --- | --- | --- | --- | --- | --- | --- | --- | --- | --- |
|  | | | | | | | | |  | | | |  | | |  | |  | |  |  | |  | |  |  | |  | |  |
| **Compound inflation index 2006** | | | | | | | | |  |  |  |  |  |  |  |  |  |  |  |  |  |  |  |  |  |  |  |  |  |  |
| 2006 | 2007 | | 2008 | | | | 2009 | | | | 2010 | | | 2011 | 2012 | | 2013 | | 2014 | | | 2015 | | 2016 | | | 2017 | | 2018 | |
| 2.334 | 2.321 | | 3.613 | | | | 2.166 | | | | 3.286 | | | 4.484 | 2.822 | | 2.555 | | 1.460 | | | 0.050 | | 0.642 | | | 2.700 | | 2.300 | |
| 100 | 102.334 | | 104.709 | | | | 108.492 | | | | 110.842 | | | 114.484 | 119.618 | | 122.993 | | 126.136 | | | 127.977 | | 128.041 | | | 128.863 | | 132.343 | |
| **Compounded inflation index 2006:** 1.323430542 | | | | | | | | | | | | | | | | | |  | |  |  | |  | |  |  | |  | |  |
|  | | | | | |  | | |  | | | |  | | |  | |  | |  |  | |  | |  |  | |  | |  |
| **Compound inflation index 2015** | | | | | | | | |  | | | |  | | |  | |  | |  |  | |  | |  |  | |  | |  |
| 2015 | | 2016 | | | | | | 2017 | | | | 2018 | | | | Inflation adjustment formulae  **Cost _Year 2_ = (Index _Year 2_ / Index _Year 1_) * Cost _Year 1_** | | | | | | | | | | | | | | |
| 0.05 | | 0.642 | | | | | | 2.700 | | | | 2.300 | | | |  |  |  |  |  |  |  |  |  |  |  |  |  |  |  |
| 100 | | 100.05 | | | | | | 100.692321 | | | | 103.4110137 | | | |  |  |  |  |  |  |  |  |  |  |  |  |  |  |  |
| **Compound inflation index 2015:** 1.034110137 | | | | | | | | | | | | | | | |  |  |  |  |  |  |  |  |  |  |  |  |  |  |  |
|  | | | | | |  | | |  | | | |  | | |  | |  | |  |  | |  | |  |  | |  | |  |
| **Compound inflation index 2017** | | | | | | | | |  | | | |  | | |  | |  | |  |  | |  | |  |  | |  | |  |
| 2017 | | | | 2018 | | | | |  | | | |  | | |  | |  | |  |  | |  | |  |  | |  | |  |
| 2.700 | | | | 2.300 | | | | |  | | | |  | | |  | |  | |  |  | |  | |  |  | |  | |  |
| 100 | | | | 102.7 | | | | |  | | | |  | | |  | |  | |  |  | |  | |  |  | |  | |  |
| **Compound inflation index 2017:** 1.027 | | | | | | | | |  | | | |  | | |  | |  | |  |  | |  | |  |  | |  | |  |
|  | | | | | |  | | |  | | | |  | | |  | |  | |  |  | |  | |  |  | |  | |  |
| **Inflation Adjusted Costs** (22,23)**:** | | | | | | | | |  | | | |  | | |  | |  | |  |  | |  | |  |  | |  | |  |
| Health state costs | | | | | Unadjusted costs | | | | | Year | | | Inflation adjusted costs (2018) | | |  | |  | | **Conversion Calculator USD to GBP** | | | | | | | | | |  |
| dmc1HA | | | | | £ 268 | | | | | 2017 | | | £ 275 | | |  | |  | | 1 USD= 0.754577 GBP (June 18th, 2017) | | | | | | | | | |  |
| dmc2HA | | | | | £ 370 | | | | | 2017 | | | £ 380 | | |  | |  | | $35,000= £26,410 | | | | | | | | | |  |
| dmcHAaft | | | | | £ 25 | | | | | 2017 | | | £ 26 | | |  | |  | |  |  | |  | |  |  | |  | |  |
| dmcHAeval | | | | | £ 53 | | | | | 2017 | | | £ 54 | | |  | |  | |  |  | |  | |  |  | |  | |  |
| dmcCI | | | | | £ 22,919 | | | | | 2018 | | | £ 22,919 | | |  | |  | |  |  | |  | |  |  | |  | |  |
| dmcCIeval | | | | | £ 4,011 | | | | | 2006 | | | £ 5,308 | | |  | |  | |  |  | |  | |  |  | |  | |  |
| dmcCIupg | | | | | £ 4,114 | | | | | 2006 | | | £ 5,445 | | |  | |  | |  |  | |  | |  |  | |  | |  |
| dmcCIcomp | | | | | £ 7,777 | | | | | 2006 | | | £ 10,292 | | |  | |  | |  |  | |  | |  |  | |  | |  |
| dmcCIaftyr1 | | | | | £ 5,000 | | | | | 2006 | | | £ 6,617 | | |  | |  | |  |  | |  | |  |  | |  | |  |
| dmcaftyr2 | | | | | £ 714 | | | | | 2006 | | | £ 945 | | |  | |  | |  |  | |  | |  |  | |  | |  |
