# Supplementary material for: Early Health Economic Modeling of Novel Therapeutics in Age-Related Hearing Loss
Source: Front Neurosci. 2022 Mar 4;16:769983. doi: 10.3389/fnins.2022.769983 (PMC8930912; doi:10.3389/fnins.2022.769983)
Supplement: Supplementary file 1 [file Data_Sheet_1.zip › SDC 8.DOCX]

| **SDC 8: Detailed description of Costs**  **Table 4.** Detailed description of Direct Costs | | | |
| --- | --- | --- | --- |
| **Description** | **Unit Costs (2018£)** | **Details** | **Reference** |
| ***Cost of novel hearing loss therapy*** |  |  |  |
| Novel Therapy Cost | £0 |  | This study |
| ***Cost of hearing aids*** |  |  |  |
| Monaural Pathway | £275 | Includes hearing aid assessment, fitting of one hearing aid device, cost of one device and the first follow-up | NHS 2017 (43) |
| Binaural Pathway | £380 | Includes hearing aid assessment, fitting of two hearing aid device, cost of two device and the first follow-up | NHS 2017 (43) |
| Cost HA aftercare | £26 | Includes repairs | NHS 2017 (43) |
| Cost of hearing evaluation for HA | £54 | Audiology hearing aid assessment only | NHS 2017 (43) |
| ***Cost of CI*** |  |  |  |
| Unilateral Cochlear Implant cost | £22, 919 | Includes the presurgical candidacy costs, cost of one device and the elective surgical costs | NHS Improvement 2018 (45) |
| Presurgical CI candidacy costs | £5,308 | Includes the presurgical candidacy costs | NHS Trust Costs  UKCISG 2004 (46) |
| ***Postimplantation costs*** |  |  |  |
| Maintenance costs in year 1 | £6,617 | Spares and repairs within the first year after surgery | NHS Trust Costs  UKCISG 2004 (46) |
| Maintenance costs in year 2+ | £945 | Spares and repairs beyond the first year after surgery. An average of the maintenance costs in years 2,3 and 4 were used. | NHS Trust Costs  UKCISG 2004 (46) |
| Processor upgrade every 5 years | £5,445 | Cost of a CI processor upgrade every 5 years. | NHS Trust Costs  Bond 2009 (47) |
| Cost of major complication | £10,292 | A major complication is defined as the necessity for reoperation after the initial CI surgery. | UKCISG 2004 (46) |
