# Supplementary material for: Early Health Economic Modeling of Novel Therapeutics in Age-Related Hearing Loss
Source: Front Neurosci. 2022 Mar 4;16:769983. doi: 10.3389/fnins.2022.769983 (PMC8930912; doi:10.3389/fnins.2022.769983)
Supplement: Supplementary file 1 [file Data_Sheet_1.zip › SDC 9.DOCX]

**SDC 9: Threshold Analysis Summary**

**Figure 1a.** Threshold Analysis Summary – Comparison of Maximum Price for different HL severities


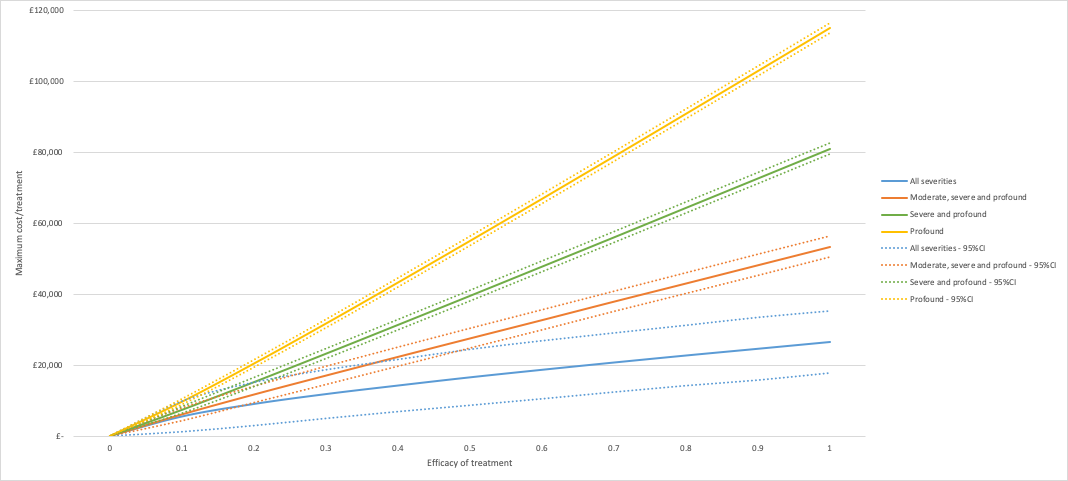


HL: hearing loss

**Figure 1b.** Threshold analysis Summary – Comparison of Number of treatments required for different HL severities


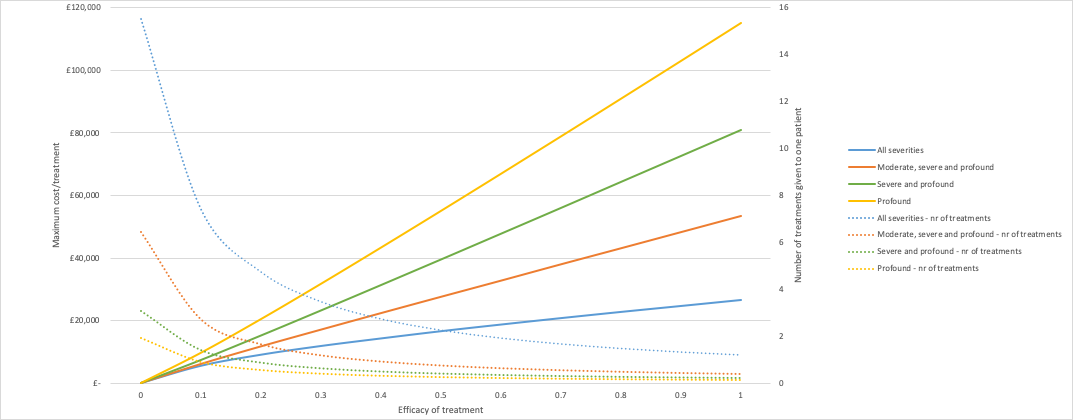


HL: hearing loss
