# Supplementary figures and images for: Early Health Economic Modeling of Novel Therapeutics in Age-Related Hearing Loss
Source: Front Neurosci. 2022 Mar 4;16:769983. doi: 10.3389/fnins.2022.769983 (PMC8930912; doi:10.3389/fnins.2022.769983)

**SDC 5: Formula to Convert Rates to Probabilities**


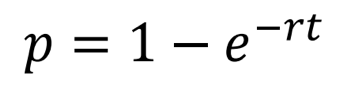


P= Probability

r= Incidence rate

t = Time

Supplement: Supplementary file 1 [file Data_Sheet_1.zip › SDC 5.DOCX]
